# Exploring attitudes toward COVID-19 vaccinations amongst healthcare workers at a national infectious disease center in Singapore—a cross-sectional study

**DOI:** 10.3389/fpubh.2026.1785244

**Published:** 2026-07-08

**Authors:** Victoria Lien, Ying Hao, Matthias Paul Han Sim Toh

**Affiliations:** 1 Preventive Medicine and Occupational Health, National University Health Systems, Singapore, Singapore; 2 National Public Health and Epidemiology Unit, National Centre for Infectious Diseases, Singapore, Singapore; 3 Department of Infectious Diseases, National Centre for Infectious Diseases and Tan Tock Seng Hospital, Singapore, Singapore; 4 Saw Swee Hock School of Public Health, National University of Singapore, Singapore, Singapore

**Keywords:** attitude to health, behavior change, COVID-19 vaccines, healthcare worker (occupational), theoretical domains framework, vaccine hesitancy

## Abstract

**Objectives:**

Vaccine hesitancy among healthcare workers (HCWs) affects pandemic preparedness and public trust. This study used the theoretical domains framework to examine COVID-19 vaccination uptake, attitudes, and information sources among staff at Singapore's National Center for Infectious Diseases (NCID).

**Methods:**

A cross-sectional anonymous electronic survey was distributed to all 695 NCID employees between July and October 2022. Participation was voluntary, and 207 staff who agreed to participate completed and submitted the survey. The questionnaire captured demographics, vaccination history and timing, intentions for future boosters, information sources, and determinants mapped to domains such as knowledge, beliefs about consequences, social role, intentions, social influences, and sentiments.

**Results:**

Of 695 staff, 207 responded (29.8%); 99.5% of the respondents had received at least one COVID-19 vaccine dose and 84.5% reported yearly influenza vaccination. Government or official sources, local news, hospital teaching, and scientific journals were key information sources. Overall, 22.7% reported reservations about COVID-19 vaccines, mainly concerning short- and long-term side effects, perceived low efficacy, and underlying medical conditions.

**Conclusion:**

COVID-19 vaccine uptake among responding HCWs was high, but nearly one-quarter still reported reservations, mainly related to side effects, perceived efficacy, and pre-existing medical conditions. Trusted institutional and official information sources, together with workplace education, appear to support vaccine confidence. These findings suggest that future vaccination strategies for HCWs should prioritize clear risk communication, trusted education channels, and approaches that support sustained voluntary booster acceptance.

## Background

Vaccine hesitancy is recognized as a multifaceted phenomenon driven by cognitive, affective, socio-economic, and structural determinants (1). Defined by the SAGE working group on vaccine hesitancy as the “*delay in acceptance or refusal of vaccines despite availability of vaccination services*” (2), the COVID-19 vaccinations presented an opportunity to explore the issue of vaccine hesitancy distinct from other established immunization programme, primarily due to the unprecedented development of the COVID-19 vaccine preparations, absence of long-term safety and efficacy data, the novelty of the mRNA technology, rapidly evolving clinical guidance and proliferation of misinformation during a period of scientific uncertainty in the pandemic.

The Theoretical Domains Framework (TDF) provides a structured behavioral model for examining factors that influence vaccination behavior, including knowledge, beliefs about consequences, intentions, social influences, reinforcement, and sentiments (3). Applying the TDF allows vaccine attitudes and behaviors to be examined systematically and helps identify modifiable barriers and enablers that can inform targeted interventions for HCWs (4).

In Singapore, the Expert Committee on COVID-19 Vaccination (EC19V) authorized the use of the BNT162b2 (Pfizer–BioNTech) messenger RNA (mRNA) vaccine in December 2020, with the national healthcare workforce prioritized in the first phase of vaccine deployment. Although initial vaccine uptake was brisk, segments of the medical community expressed concern over the safety profile, efficacy, and long-term effects of the novel vaccines (5). The subsequent emergence of variants of concern exhibiting immune escape mechanisms (6, 7), coupled with evidence of waning immunity (8, 9), prompted policy adjustments. A booster immunization programme was implemented in September 2021. While complete primary-series vaccination was mandated for HCWs from 1 October 2021, booster doses remained strongly recommended but voluntary. In 2022, Singapore faced two successive Omicron subvariant waves, the BA.4/BA.5 and XBB subvariant, with a peak 7-day moving average of approximately 18,300 daily infections, and hospitalized cases peaking at over 800 on a single day. Reinfections were also increasing, comprising approximately 17% of new cases by October 2022 (10). HCWs from the National Center for Infectious Diseases (NCID), the designated national infectious diseases center, were at the frontline of Singapore's national response to COVID-19 and continued to face continuous and direct occupational exposure to COVID-19, both in the laboratory and clinical settings. Given the changing pandemic context, continued occupational exposure, and the shift from mandatory primary vaccination to voluntary booster uptake, understanding HCWs' attitudes toward COVID-19 vaccination remained necessary in this setting.

## Objectives

This study aims to examine COVID-19 vaccination uptake in NCID's HCWs and the factors that encouraged or delayed vaccine acceptance. This study's secondary objective is to understand the sources of information of COVID-19 vaccinations relied upon by HCWs in NCID.

## Methods

### Study population, means of data collection

A cross-sectional study was conducted among all 695 HCWs employed at NCID. A self-administered anonymous electronic survey was distributed between 18 July and 25 October 2022 through email, ward-based chat groups, and hospital teaching sessions (Supplementary Material A). Participation was voluntary. The survey was completed only by staff who agreed to participate and submitted their responses. A total of 207 completed responses were received and included in the analysis.

The survey was hosted on FormSG, a secured encrypted cloud-based platform used by Singapore government and public institutions. No personal identifiers were collected. Respondents accessed the survey through a standard URL or QR code. The data collected from each response was stored on FormSG's secure database.

### Measurements

The survey collected information on participants' (i) Basic demographic data and job scope, (ii) Knowledge and attitudes toward COVID-19 vaccinations, (iii) Access to COVID-19 information and, (iv) Vaccine uptake practices and intentions toward COVID-19 vaccines.

Demographic data collected included age group, department and job group, gender, number of vaccination doses received, speed of the first dose of the COVID-19 vaccine uptake (by date range) and occupational exposure to COVID-19.

The survey instrument was designed using domains from the Theoretical Domains Framework. The domains that were incorporated include knowledge, beliefs about consequences, reinforcement, social/ professional role and identity, intentions, social influences, and sentiments. Key constructs were measured using a 4-point Likert Scale, “*Yes*” or “*No*” responses, choosing between multiple predicted responses, and free text where appropriate.

i) *Knowledge* was assessed by asking respondents to indicate their sources of information, access to teaching, and quality of the teaching received, and their confidence and regularity in assessing sources for accuracy and bias.
ii) *Beliefs about consequences* was assessed by asking respondents to indicate motivators (multiple responses allowed) for vaccination and to list their top reservations against the COVID-19 vaccine.
iii) *Reinforcement* was assessed by surveying respondents' exposure to patients with severe COVID-19 disease and history of influenza vaccination.
iv) *Social or professional role and identity* was examined by comparing the responses by job groups.
v) *Intention* was assessed through the speed of the first COVID-19 vaccine uptake and acceptance of future doses or variant specific boosters.
vi) *Social Influence* was measured by examining the proportion of respondents who chose “*pressure from family*,” “*pressure from friends*,” “*pressure from workplace*,” “*vaccine differentiated measures*” as their motivation for being vaccinated. Vaccine-differentiated measures refer to Singapore's Vaccination-Differentiated Safe Management Measures (VDS), a unique government policy framework implemented from late-2021 onwards as part of the nationwide strategy in lifting social restrictions. There were differential restrictions based on vaccination status, whereby only fully vaccinated individuals were permitted to participate in social events and large gatherings. VDS were extended to the workplace, including hospitals, such that only vaccinated HCWs were permitted to return to the workplace, with unvaccinated employees required to undergo regular testing at their own expense and during their own time, creating a tangible occupational consequence for remaining unvaccinated within the workplace (11).
vii) *Sentiments* were assessed by two items consistent with the framework described by Larson et al. (12). Fear of COVID-19 on a 4-point Likert Scale (1 = “Not afraid at all,” 2 = “A bit afraid,” 3 = “Afraid” and 4 = “Very afraid”) and reception to COVID-19 vaccines on a binary response (“*overall positive*,” “*overall negative*”).

### Data analysis

Qualitative analysis was limited to selected questions. Free-text responses for open-ended questions were consolidated and coded into themes based on the Theoretical Domains Framework. The free-text responses were collected from Question B6 (see Supplementary Material B) and independently coded by two members of the research team. Given the small number of responses, we were unable to stratify the data into distinct thematic domains. Instead, we conducted an inductive content analysis to identify recurring concepts. The most frequently noted keywords included “side effects,” “poor efficacy,” “medical conditions,” “drug approval process,” and “novel.” Two responses were additionally classified as reflecting an overall negative perception when participants expressed general distrust or uncertainty without specifying a clear reason.

Quantitative analysis was performed using R 4.1.2. Descriptive analysis included calculating frequency and proportions. The correlation between 2 variables was evaluated using a chi square test or Fisher's exact test when more than 20% of cells have expected counts less than 5 in the contingency tables. We also calculated Cramér's V which quantifies the strength of association between categorical variables. *p* value < 0.05 was treated as statistically significant.

## Results

The response rate for the survey was 29.8%, with 207 responses received from 695 NCID HCW. The highest response rate of 65.6% was from Public Health and Research departments, and the lowest response rate of 10.0% from nursing. Overall, 99.5% of all staff received at least 1 Covid-19 vaccine dose (Table 1).

**Table 1:** Characteristics of respondents.

	Number of respondents	
	*n* = 207 no. (%)
Age group (years)	
21–29	60 (29.0)
30–39	78 (37.7)
40–49	42 (20.3)
50–59	20 (9.70)
60+	7 (4.3)
Gender	
Female	151 (72.9)
Male	56 (27.1)
Job group	
Administrative/operations	62 (30.0)
Allied health	42 (20.3)
Medical	29 (14.0)
Nursing	32 (15.5)
Public health/research	42 (20.3)

### Knowledge

Overall, 23.2% of respondents indicated that their friends and family were a source of information regarding COVID-19 vaccinations, while 37.2% indicated using local new sources. 22.2% sourced information from social media, 66.2% from government or official sources of information and 38.6% from scientific journals or other academic sources. 51.7% of respondents indicated relying on hospital teaching and sharing and 43.5% indicated industry or professional sources (including MOH circulars) as a source of information (Table 2).

**Table 2:** Knowledge about COVID-19 Vaccines and Information Sources.

	*N*	Job group					*p*-value^b^	Cramér's V^c^
		Administration/Ops	Allied health	Medical	Nursing	Public health/research		
		*n* = 62 no. (%)	*n* = 42 no. (%)	*n* = 29 no. (%)	*n* = 32 no. (%)	*n* = 42 no. (%)		
Sources of information								
Friends or family	48 (23.2)	17 (27.4)	10 (23.8)	8 (27.6)	5 (15.6)	8 (19.0)	0.67	0.107
Local news sources	130 (62.8)	48 (77.4)	31 (73.8)	12 (41.4)	14 (43.8)	25 (59.5)	**0.001**	0.301
Social media (facebook, tiktok, instagram, telegram)	46 (22.2)	18 (29.0)	11 (26.2)	5 (17.2)	3 (9.4)	9 (21.4)	0.24	0.164
Government or official sources of information	137 (66.2)	43 (69.4)	29 (69.0)	11 (37.9)	22 (68.8)	32 (76.2)	**0.013**	0.248
Scientific journals or other academic sources	80 (38.6)	10 (16.1)	17 (40.5)	20 (69.0)	10 (31.2)	23 (54.8)	**< 0.001**	0.380
Hospital teaching or sharing	107 (51.7)	24 (38.7)	23 (54.8)	14 (48.3)	23 (71.9)	23 (54.8)	**0.043**	0.218
Industry or professional Sources, MOH circulars	90 (43.5)	22 (35.5)	20 (47.6)	12 (41.4)	13 (40.6)	23 (54.8)	0.375	0.143
Do you regularly assess sources of information for bias and accuracy?								
No	66 (31.9)	24 (38.7)	14 (33.3)	5 (17.2)	12 (37.5)	11 (26.2)	0.256	0.160
Yes	141 (68.1)	38 (61.3)	28 (66.7)	24 (82.8)	20 (62.5)	31 (73.8)		
Do you feel confident to assess sources of information for bias and accuracy?								
No	51 (24.6)	18 (29)	12 (28.6)	4 (13.8)	8 (25)	9 (21.4)	0.547	0.122
Yes	156 (75.4)	44 (71)	30 (71.4)	25 (86.2)	24 (75)	33 (78.6)		
Have you attended any talks or engagement sessions regarding COVID-19 vaccines?								
No	85 (41.1)	29 (46.8)	20 (47.6)	6 (20.7)	13 (40.6)	17 (40.5)	0.096	0.187
Yes (not helpful)	11 (5.3)	2 (3.2)	3 (7.1)	1 (3.4)	0 (0)	5 (11.9)		
Yes (helpful)	109 (52.7)	30 (48.4)	18 (42.9)	22 (75.9)	19 (59.4)	20 (47.6)		
Did NCID do anything to improve your understanding of the safety and efficacy of COVID-19 vaccines?								
No	51 (24.6)	16 (25.8)	8 (19.0)	10 (34.5)	5 (15.6)	12 (28.6)	0.675	0.121
Yes	137 (66.2)	39 (62.9)	29 (69.0)	18 (62.1)	25 (78.1)	26 (61.9)		
Others^a^	19 (9.2)	7 (11.3)	5 (11.9)	1 (3.4)	2 (6.2)	4 (9.5)		

^a^*Maybe, unsure*; ^b^*By Chi-square tests*; ^c^*Only Local News Sources and Scientific Journals or other Academic Sources show medium to large association with job group while other variables had weak association with job group*.

There was a statistically significant difference between staff of various job groups and utilizing local news sources (*p* = 0.001), government or official sources (*p* = 0.013), scientific journals or other academic sources (*p* < 0.001), and hospital teaching or sharing (*p* = 0.042). There was no association found for the other sources of information for COVID-19 vaccines. Allied Health, Administrative and operations staff tended to rely on local news sources or government sources of information (69–73%), while 76.2% of Public Health/Research Staff relied on Government or official sources. 71.9% of nurses reported relying on hospital teaching. 69% of Medical Staff reported relying on scientific journals and were less reliant on government or official sources of information (Table 2).

66.7% of staff perceived that NCID made attempts to improve their knowledge of COVID-19 vaccines. Of those who attended hospital or Ministry of Health organized information sessions, 90.8% of them reported that they were helpful. 41.1% of respondents indicated non-attendance at any teaching or engagement sessions for COVID-19 vaccines. 5.3% responded that they attended sessions but found them unhelpful. 52.7% reported attending teaching sessions and found them helpful. There was no association for attendance by age or job group (Table 2).

To explore the potential influence of misinformation on vaccine acceptance in HCWs, respondents were asked if they assessed content for accuracy and bias and felt confident doing so. 61.8% of respondents reported regularly assessing sources of information for bias and accuracy, and 75.4% reported doing so regularly. There was no association found between source appraisal and age or job group (Table 2).

### Beliefs about consequences

42 respondents listed their reservations about COVID-19 vaccines as free text. 59.5% of responses were concerns about short- and long-term side effects. 14.3% of responders perceived that the vaccine had low efficacy, 9.5% of respondents were concerned about their past medical conditions being aggravated by the vaccine, 7.1% had low confidence in the drug approval process, 4.8% cited the novel nature of the vaccine and 4.8% had an overall poor perception of the vaccine (Table 3).

**Table 3:** Reservations about COVID-19 vaccines.

Reservations about the COVID-19 vaccine^#^	Frequency of responses
	*n* = 42 No. (%)
Concern about short- and long-term side effects	25 (59.5)
Perception of poor vaccine efficacy	6 (14.3)
Past medical conditions	4 (9.5)
Low confidence in drug approval process	3 (7.1)
Novel nature of the vaccine	2 (4.8)
Poor perception overall	2 (4.8)

^#^*Coded by key phrases/themes*.

### Social influence and social/ professional role and identity

When asked whether they had any reservations about COVID-19 vaccines, 22.7% (47 people) responded “*yes*.” There was no association between having COVID-19 vaccine reservations and age or job group (Table 4).

**Table 4:** Reservations about COVID-19 Vaccines by Job and Age Group.

	*N*	Job group					*p*-value^b^	Cramér's V^c^
		Administration/operation	Allied health	Medical	Nursing	Public health/research		
		*n* = 62 no. (%)	*n* = 42 no. (%)	*n* = 29 no. (%)	*n* = 32 no. (%)	*n* = 42 no. (%)		
Did you have any reservations about getting COVID-19 vaccines?								
No	160 (77.3)	48 (77.4)	31 (73.8)	25 (86.2)	25 (78.1)	31 (73.8)	0.753	0.096
Yes	47 (22.7)	14 (22.6)	11 (26.2)	4 (13.8)	7 (21.9)	11 (26.2)		
Age group (years old)								
	***N***	**20**–**29**	**30**–**39**	**40**–**49**	**50**–**59**	**60+**	***p*** **-value** ^**b**^	**Cramér's V** ^**c**^
		***n*** **=** **60 no. (%)**	***n*** **=** **78 no. (%)**	***n*** **=** **42 no. (%)**	***n*** **=** **20 no. (%)**	***n*** **=** **7 no. (%)**		
Did you have any reservations about getting COVID-19 vaccines?								
No	160 (77.3)	46 (76.7)	62 (79.5)	33 (78.6)	13 (65.0)	6 (85.7)	0.733	0.105
Yes	47 (22.7)	14 (23.3)	16 (20.5)	9 (21.4)	7 (35.0)	1 (14.3)		

^*b*^*By Chi-square tests;* ^*c*^*Both Cramér's V indicate weak association*.

Participants were asked to indicate their main motivators for receiving COVID-19 vaccines. Multiple responses were allowed. 77.3% of respondents did not indicate any reservation to COVID-19 vaccines, and the top 3 motivators were “*fear of illness or disease”* (61.3%), “*confidence in the drug approval process and safety profile”* (70.0%) and “*better understanding of the disease”* (63.3%). For those who had reservations against COVID-19 vaccinations, the top 3 motivators were “*pressure from the workplace” (61.7%)*, “*vaccine differentiated measures”* (40.4%) and “*better understanding of vaccines”* (38.3%).

“*Pressure from friends”* and “*pressure from family”* were not found to be strong motivators for vaccination in either group. Among those with reservations about vaccination (22.7%), 4.3% reported being vaccinated due to pressure from friends, while none reported being vaccinated due to pressure from family. Among those without reservations, 1.2% reported being vaccinated due to pressure from friends, and 2.5% owing to pressure from family (Table 5).

**Table 5:** Motivations for being vaccinated against COVID-19.

Motivations for being vaccinated against COVID-19	Reservations about Covid-19 vaccines	
	Yes	No
	*n* = 47 no. (%)	*n* = 160 no. (%)
Fear of illness/disease	14 (29.8)	98 (61.3)
Confidence in drug approval process/safety profile	11 (23.4)	112 (70.0)
Information: better understanding of vaccines	18 (38.3)	102 (63.7)
Pressure from workplace	29 (61.7)	27 (16.9)
Pressure from friends	2 (4.3)	2 (1.2)
Pressure from family	0 (0)	4 (2.5)
Vaccine differentiated measures	19 (40.4)	35 (21.9)
Others	7 (14.9)	8 (5.0)

### Reinforcement

Overall, 85.5% of respondents reported receiving yearly influenza vaccination, with coverage highest among nursing staff (100.0%) and medical staff (86.2%), and lowest among administrative and operations staff (77.4%). There was a statistically significant difference in regular influenza vaccination by job group (*p* = 0.028), but not by age group. In addition, 25.1% of respondents reported knowing an individual (family, friends, or patients) who had severe COVID-19 disease prior to vaccine availability, with higher proportions among medical (51.7%) and nursing staff (40.6%) compared to other job groups. Exposure to someone with severe COVID-19 disease differed significantly by job group (*p* = 0.001).

### Intention

36.7% of respondents reported registering for their first COVID-19 vaccine dose < 1 week from eligibility. 33.3% reported registering between 1 week and 1 month, 20.3% between 1 and 3 months, and 4.8% between 3 and 6 months from eligibility. 3.9% of respondents reported waiting 6 or more months to register. There was no significant difference in the time to registration by job or age groups (Table 6).

**Table 6:** Time to Registration for first COVID-19 Vaccine by Age and Job Group.

	*Age group (Years)*					*p*-value	Cramér's V^c^	*Job group*					*p*-value	Cramér's V^c^
	20–29	30–39	40–49	50–59	60+			Administration/operations	Allied health	Medical	Nursing	Public health/research		
	*n* = 60 no. (%)	*n* = 78 no. (%)	*n* = 42 no. (%)	*n* = 20 no. (%)	*n* = 7 no. (%)			*n* = 62 no. (%)	*n* = 42 no. (%)	*n* = 29 no. (%)	*n* = 32 no. (%)	*n* = 42 no. (%)		
Time to register for COVID-19 vaccination (from date of eligibility)														
< 1 week	20 (33.3)	30 (38.5)	16 (38.1)	7 (35.0)	3 (42.9)	0.928	0.119	17 (27.4)	15 (35.7)	19 (65.5)	9 (28.1)	16 (38.1)	0.078	0.192
1 week −1 month	20 (33.3)	23 (29.5)	14 (33.3)	9 (45.0)	3 (42.9)			20 (32.3)	16 (38.1)	7 (24.1)	14 (43.8)	12 (28.6)		
3 months	16 (26.7)	15 (19.2)	8 (19.0)	3 (15.0)	0 (0)			14 (22.6)	7 (16.7)	3 (10.3)	6 (18.8)	12 (28.6)		
3–6 months	2 (3.3)	6 (7.7)	1 (2.4)	1 (5.0)	0 (0)			4 (6.5)	4 (9.5)	0 (0)	2 (6.2)	0 (0)		
>6 months	2 (3.3)	3 (3.8)	2 (4.8)	0 (0)	1 (14.3)			5 (8.1)	0 (0)	0 (0)	1 (3.1)	2 (4.8)		
NA^a^	0 (0)	1 (1.3)	1 (2.4)	0 (0)	0 (0)			2 (3.2)	0 (0)	0 (0)	0 (0)	0 (0)		

^*a*^*Not fully vaccinated;* ^*b*^*By Chi-square tests, for a 6x5 table;* ^c^*Cramér's V 0.119 indicates a weak association, 0.192 medium to large association*.

Overall, 67.1% of respondents indicated that they would accept yearly COVID-19 vaccine booster doses, and 74.4% of respondents indicated accepting variant-specific booster vaccinations. There was an association between age and acceptance of variant specific vaccinations (*p* = 0.013), with the 40–49-year-old age group being the least accepting of variant specific vaccines (31.0% indicated “no”). In contrast, 15.0% of 21–29-year-old, 7.7% of 30–39-year-old, 15.0% of 50–59-year-old and 28.6% of 60 years or older respondents indicated non-acceptance of variant specific boosters. There was no association between acceptance of yearly COVID-19 boosters by age or job group, or of variant-specific vaccines by job group (Table 7).

**Table 7:** Association between intentions towards COVID-19 vaccines by age group.

	Age group (years old)						
	20–29	30–39	40–49	50–59	60+	*p*-value^b^	Cramér's V^c^
	*n* = 60 no. (%)	*n* = 78 no. (%)	*n* = 42 no. (%)	*n* = 20 no. (%)	*n* =7 no. (%)		
Would you accept receiving yearly COVID-19 booster vaccinations, if necessary?							
No	10 (16.7)	13 (16.7)	12 (28.6)	4 (20.0)	2 (28.6)	0.329	0.140
Yes	40 (66.7)	58 (74.4)	26 (61.9)	12 (60.0)	3 (42.9)		
Others ^a^	10 (16.7)	7 (9.0)	4 (9.5)	4 (20.0)	2 (28.6)		
Would you accept receiving COVID-19 *variant specific* booster vaccinations, if necessary?							
No	9 (15.0)	6 (7.7)	13 (31.0)	3 (15.0)	2 (28.6)	**0.013**	0.209
Yes	44 (73.3)	68 (87.2)	23 (54.8)	14 (70.0)	5 (71.4)		
Others ^a^	7 (11.7)	4 (5.1)	6 (14.3)	3 (15.0)	0 (0)		

^a^*3rd option: “unsure, depends, maybe”;* ^*b*^*By Chi-square tests, for a 3x5 table;* ^*c*^*Cramér's V 0.14 indicates a weak association while 0.209 a medium association*.

There was no association between the degree of fear of COVID-19 and regular influenza vaccine uptake, knowing anyone with severe COVID-19 disease, and acceptance of regular future COVID-19 vaccines (Table 8).

**Table 8:** Association between degree of fear of COVID-19 and intentions towards COVID-19 vaccines.

	Degree of fear of COVID-19 prior to vaccine availability					
	Not afraid at all	A bit afraid	Afraid	Very afraid	*p*-value^b^	Cramér's V^c^
	*n* = 43 no. (%)	*n* = 98 no. (%)	*n* = 50 no. (%)	*n* = 16 no. (%)		
Do you receive influenza vaccines yearly?						
No	8 (18.6)	13 (13.3)	7 (14.0)	4 (25.0)	0.532	0.095
Yes	35 (81.4)	85 (86.7)	43 (86.0)	12 (75.0)		
Did anyone you know (including family, friends, patients) have severe COVID-19 disease?						
No	33 (76.7)	72 (73.5)	38 (76.0)	12 (75.0)	0.983	0.032
Yes	10 (23.3)	26 (26.5)	12 (24.0)	4 (25.0)		
Would you accept receiving yearly COVID-19 booster vaccinations, if necessary?						
No	7 (16.3)	21 (21.4)	9 (18.0)	4 (25.0)	0.732	0.094
Yes	31 (72.1)	67 (68.4)	31 (62.0)	10 (62.5)		
Others^a^	5 (11.6)	10 (10.2)	10 (20.0)	2 (12.5)		
Would you accept receiving COVID-19 variant specific booster vaccinations, if necessary?						
No	7 (16.3)	16 (16.3)	6 (12.0)	4 (25.0)	0.722	0.090
Yes	33 (76.7)	74 (75.5)	37 (74.0)	10 (62.5)		
Others^a^	3 (7.0)	8 (8.2)	7 (14)	2 (12.5)		

^a^*3rd option: “unsure, depends, maybe”;* ^*b*^*By Chi-square tests;* ^*c*^*All Cramér's V values indicate weak association*.

### Sentiments

88.4% of respondents reported “*overall positive”* reactions to the COVID-19 vaccine being made available in Singapore. Younger age groups had a more positive perception of the vaccine. 96.7% of 21–29-year-olds, 89.7% of 30–39-year-olds, 81.0% of 40–49-year-olds, 80% of 50–59 year–olds and 71.4% of 60 years or older respondents reported having an “overall positive” perception of the vaccine. The positive sentiments of the vaccine declined with age (*p* = 0.028).

When rating their fear of COVID-19 prior to vaccine availability, 20.8% of respondents reported being “*not afraid at all,”* 47.3% reported being “a bit afraid.” 24.2% reported being “*afraid”* and 7.7% being “*very afraid,”* Degree of fear of COVID-19 was different between job groups (*p* = 0.01), but not by age groups (Table 9).

**Table 9:** Association between degree of fear of COVID-19 and exposure to the virus before and after vaccine availability, age groups, gender and job groups.

	Fear of Covid-19 prior to vaccine availability					
	Not afraid at all	A bit afraid	Afraid	Very afraid	*p*-value^b^	Cramér's V^c^
	*n* = 43 no. (%)	*n* = 98 no. (%)	*n* = 50 no. (%)	*n* = 16 no. (%)		
Did you have exposure to COVID-19 through patient contact or laboratory samples, before the COVID-19 vaccine was available?						
Yes	25 (58.1)	57 (58.2)	40 (80.0)	13 (81.2)	**0.021**	0.217
No	18 (41.9)	41 (41.8)	10 (20.0)	3 (18.8)		
Did you have exposure to COVID-19 through patient contact or laboratory samples, after the COVID-19 vaccine was available?						
Yes	21 (48.8)	45 (45.9)	33 (66.0)	10 (62.5)	0.101	0.173
No	22 (51.2)	53 (54.1)	17 (34.0)	6 (37.5)		
Age group (years old)						
21–29	14 (32.6)	24 (24.5)	17 (34.0)	5 (31.2)	0.73	0.112
30–39	16 (37.2)	42 (42.9)	16 (32.0)	4 (25.0)		
40–49	8 (18.6)	20 (20.4)	10 (20.0)	4 (25.0)		
50–59	2 (4.7)	10 (10.2)	6 (12.0)	2 (12.5)		
60+	3 (7.0)	2 (2.0)	1 (2.0)	1 (6.2)		
Gender						
Female	24 (55.8)	71 (72.4)	42 (84.0)	14 (87.5)	**0.012**	0.233
Male	19 (44.2)	27 (27.6)	8 (16.0)	2 (12.5)		
Job group						
Administration/operations	6 (14.0)	29 (29.6)	18 (36.0)	9 (56.2)	**0.009**	0.200
Allied health	7 (16.3)	17 (17.3)	13 (26.0)	5 (31.2)		
Medical	7 (16.3)	19 (19.4)	2 (4.0)	1 (6.2)		
Nursing	10 (23.3)	14 (14.3)	7 (14.0)	1 (6.2)		
Public health/research	13 (30.2)	19 (19.4)	10 (20.0)	0 (0)		

^*b*^*By Chi-square tests*.

An association was found between degree of fear of COVID-19 and having occupational exposure to the virus prior to vaccine availability (*p* = 0.021). However, there was no association found between fear and occupational exposure to the virus *after* the vaccine was made available. There was no association between fear of COVID-19 and time taken to register for both the primary series and booster doses (Table 9).

## Discussion

While numerous studies have examined COVID-19 vaccine intentions among healthcare workers in diverse settings, most have focused on early pandemic phases, primary-series vaccination, or generic attitudinal determinants, with relatively fewer studies using a behavioral framework to characterize determinants across different professional groups in later pandemic waves.

By applying the Theoretical Domains Framework to staff at Singapore's National Center for Infectious Diseases during successive Omicron waves, this study examined how knowledge, beliefs about consequences, social/professional role, reinforcement, social influences, and sentiments may jointly shape vaccine reservations and future booster intentions in a highly exposed workforce, operating under mandatory primary vaccination but voluntary boosters. These findings address a gap in the literature by exploring context-specific determinants of continued COVID-19 vaccine engagement among HCWs in the later phases of the pandemic and in settings with strong policy levers such as vaccine-differentiated measures.

### Summary of key findings

To promote vaccination update, access to reliable information about COVID-19 vaccines should be provided by the workplace. 66.7% of staff perceived that NCID made attempts to improve their knowledge of COVID-19 vaccines. Of those who attended teaching sessions, 90.8% felt that teaching sessions were helpful. About 2 in 5 respondents reported not attending any teaching or information sessions on COVID-19 vaccines (whether conducted by NCID or other agencies). Ensuring that HCWs have access to accurate and reliable information about COVID-19 vaccines remains essential. The fact that a significant proportion of HCWs reported not participating in any educational sessions highlights an important gap that can be easily addressed. Moving forward, targeted strategies to engage all healthcare staff and involve them in vaccine campaigns could enhance vaccine uptake and strengthen public health initiatives.

This study also explored the potential influence of misinformation on vaccine acceptance. 68.1% of respondents reported appraising sources for accuracy and bias, and 75.4% of them reported feeling confident doing so. When searching for COVID-19 vaccine information, the majority of NCID's HCWs generally relied on more reliable sources of information, while a lower proportion of respondents relied on social media (22.2%) and friends or family (23.2%). Allied Health, Administrative and operations staff tended to rely on local news sources or government sources of information (69% and 73% respectively). 76.2% of Public Health/ Research Staff relied on Government or official sources. 71.9% of nurses reported relying on hospital teaching and 69% of Medical Staff reported relying on scientific journals and were less reliant on government or official sources of information. While it was not possible to conclusively interpret that NCID HCWs' access to reliable information had led to a higher vaccination uptake, a randomized controlled study by Loomba et al. found that access to misinformation to COVID-19 lowered COVID-19 vaccine acceptance and vice versa (13). Hence, given that the majority of the respondents relied on various more reliable sources of information relating to COVID-19 vaccinations, this was a promising finding that suggests misinformation to COVID-19 may not be a major determinant in the vaccine uptake amongst NCID HCWs.

To better understand the factors that deterred or promoted vaccine uptake in this unique population, this study asked participants to list their reservations and motivators for being vaccinated against COVID-19. The top 3 reservations about COVID-19 vaccines reported by study participants were (1) short- and long-term side effects, (2) perception that the vaccine had low efficacy, and (3) concerns about their past medical conditions being aggravated by the vaccine. While these findings were limited to 42 respondents, responses were in keeping with the negative influencers found by Huynh et al. and others (14, 15). The top 3 motivators for vaccination both in general, and for those who had no reservations about COVID-19 vaccines were similar to findings of the rapid review conducted by Crashaw and her colleagues in Canada (16). These were “*fear of illness or disease”* (61.3%), “*confidence in the drug approval process and safety profile”* (70.0%) and “*better understanding of the disease”* (63.3%).

As the primary COVID-19 series vaccination was mandated for all NCID HCWs, the study finding of 99.5% uptake of least 1 COVID-19 vaccination dose reflects near-complete institutional compliance rather than voluntary vaccine acceptance. While pressure from institutional policies and vaccine-differentiated measures may be effective early in the pandemic or as a strategy to compel or motivate those who are possibly vaccine-hesitant, pressure tactics are never desirable in the first instance as this may not lead to meaningful and sustained vaccine engagement later in the pandemic. The study findings suggest that perhaps as an alternative, early education efforts for vaccine initiatives can focus and emphasize on drug safety and efficacy, which are worthwhile pursuits for healthcare organizations, even before such vaccines are made locally available. The other social influence domain factors included in the survey, “*Pressure from friends”* and “*pressure from family.”* were not found to be strong motivators for vaccination. Reasons for this are uncertain and should be explored further.

Findings from this study suggest that individuals over 50 years old may be more likely to have an overall negative perception of the COVID-19 vaccine, though interpretation of this data was limited by low numbers of participants over the age of 50 and contradict the findings by multiple studies which found greater COVID-19 vaccine acceptance for older age groups (1, 17, 18).

Interestingly, the study found that degree of fear of contracting COVID-19 was found to be different between job groups (*p* = 0.01), but not by age groups. This could be explained by the association found between degrees of fear of COVID-19 and having occupational exposure to the virus prior to vaccine availability (*p* = 0.021). Heightened occupational exposure clearly increases risk of disease. That no association was found between fear and occupational exposure to the virus, *after* the vaccine was made available further supports that fear of disease and perceived disease severity can promote the uptake of novel vaccinations in exposed populations.

Considering the substantial literature that lists access to information and understanding of COVID-19 vaccines as an enabling factor for vaccination (15, 19, 20), more efforts can be made to increase workplace attendance of information sessions. Findings from this study suggest that the following areas may be prioritized when designing staff communications for COVID-19 vaccine information–(i) Vaccine Safety and Efficacy, (ii) Possible side effects and risk factors, (iii) Consequences of infection and transmission (fear of COVID-19) and, (iv) Explaining the drug approval, licensing, and development process.

Finally, while this study does not evaluate the impact of HCWs on community attitudes toward vaccination, the authors recognize their potential influence and emphasizes the importance of ensuring that HCWs are properly informed. The findings illustrate potential gaps in access to reliable vaccine information and participation in educational sessions, suggesting the need for targeted efforts to engage and educate healthcare personnel. By investing in HCWs' education and actively involving them in vaccine campaigns, there is an opportunity to leverage their respected status to address vaccine hesitancy and improve public health outcomes.

### Strengths and limitations

This study has several strengths. It is the first to formally examine access to, and use of, COVID-19 vaccine information sources among healthcare workers in Singapore, using a behavioral framework tailored to HCWs. The survey was also conducted in a national infectious disease center during successive Omicron waves, amongst staff with high occupational exposure and direct involvement in Singapore's COVID-19 response. This provides context specific information into determinants of vaccine reservations and booster intentions in a high-risk setting. By mapping items to multiple domains of the Theoretical Domains Framework, including knowledge, beliefs about consequences, reinforcement, social/professional role and identity, intentions, social influences and sentiments, the study offers a more nuanced characterization of behavioral determinants than generic attitude surveys. Finally, the inclusion of diverse job groups (administration/operations, allied health, medical, nursing, public health/research) allowed comparison of information-seeking patterns, previous exposure, and intentions across professional roles, which is useful for tailoring workplace communication strategies.

### Measurement and instrument limitations

The survey instrument was purpose-built due to the absence, at the time, of a validated tool specifically designed to assess COVID-19 vaccine hesitancy in HCWs using the TDF, and it was not formally piloted or cognitively pre-tested prior to deployment. Although items were informed by a validated TDF-based framework used in a rapid review of HCWs' vaccine hesitancy (14), some constructs were represented by proxy measures and not all TDF domains were included, potentially limiting construct validity and the completeness of behavioral determinants captured.

### Question framing and outcome measurement

In questions about future boosters, the phrase “if necessary” may have been interpreted as implying future governmental or institutional requirements rather than voluntary choice, which could overestimate true willingness to accept boosters. In addition, booster uptake was not systematically collected, so we were unable to triangulate stated intentions with actual booster behavior in the post-mandate period.

### Sampling, selection and non-response bias

Several sampling and recruitment issues also limit the interpretation of our findings. Contract staff (e.g. cleaners, security personnel) and certain staff groups (healthcare associates, patient service associates) were not included, despite their exposure and potential vulnerability, because of restricted access during safe-distancing measures. In addition, using hospital teaching sessions both as a recruitment channel and as an exposure of interest likely introduced selection bias, because staff who attend teaching are more engaged with institutional education and may be more positively disposed toward hospital messaging; this could overestimate both reliance on and satisfaction with teaching. The overall response rate of 29.8%, and particularly the low response among nurses (10.0%), further suggests that our respondents are a self-selected subset of more engaged and pro-vaccination staff, such that vaccine hesitancy and reservations among more hesitant HCWs are probably under-represented.

### Generalizability

The study findings are most applicable to staff working in a tertiary national infectious diseases center in Singapore. The low overall response rate, under-representation of nursing staff, and exclusion of contract and ancillary staff mean that our results likely reflect the attitudes and behaviors of a more engaged and pro-vaccination subset of NCID staff, rather than the full HCW population. Extrapolating these findings to other institutions, jurisdictions without similar policy levers, or to older, less formally educated or non-clinical workers should thus be made with caution. Nevertheless, the behavioral patterns identified, including the importance of trusted institutional and official information sources, successfully allaying concerns over safety and efficacy among those with reservations, and addressing differing motivators between hesitant and non-hesitant staff, are likely to be relevant to other high-risk healthcare settings seeking to sustain voluntary booster uptake as pandemic conditions and vaccine policies evolve.

## Conclusions

Among responding NCID HCWs, COVID-19 vaccine uptake was high and willingness to receive future boosters was generally favorable, although a meaningful minority reported reservations related mainly to safety, efficacy, and pre-existing medical conditions. Trusted official information sources and workplace education were commonly reported enablers, while workplace requirements were prominent motivators among those with prior reservations. These findings support targeted and credible communication strategies to sustain voluntary vaccine confidence and booster acceptance among HCWs.

## Data Availability

The original contributions presented in the study are included in the article/Supplementary material, further inquiries can be directed to the corresponding author.
