# Supplementary material for: Exploring attitudes toward COVID-19 vaccinations amongst healthcare workers at a national infectious disease center in Singapore—a cross-sectional study
Source: Front Public Health. 2026 Jul 8;14:1785244. doi: 10.3389/fpubh.2026.1785244 (PMC13388545; doi:10.3389/fpubh.2026.1785244)
Supplement: Supplementary file 1 [file Data_Sheet_1.PDF]

COVID-19 Vaccine Uptake Rates in A High-Risk Population - Health Behaviours of Staff in the National Centre for Infectious Diseases (NCID)

**Section A: Demographic information**	
1. Gender	☐ Male<br>☐ Female
2. Age (years)	☐ 21-29<br>☐ 30-39<br>☐ 40-49<br>☐ 50-59<br>☐ 60+
3. Number of mRNA COVID Vaccines received	☐ 0<br>☐ 1<br>☐ 2<br>☐ 3<br>☐ >3
4. Number of non-mRNA COVID Vaccines received	☐ 0<br>☐ 1<br>☐ 2<br>☐ 3<br>☐ >3
5. Do you receive Influenza vaccination annually?	☐ Yes<br>☐ No
6. Which best describes your Job Group?	☐ Clinical: Medical<br>☐ Clinical: Nursing<br>☐ Clinical: Allied Health<br>☐ Administrative / Operations<br>☐ Public Health Units / Research
7. Did you have exposure to COVID-19 through patient contact or laboratory samples, *before* the COVID-19 vaccine was available?	☐ Yes<br>☐ No
8. Did you have exposure to COVID-19 through patient contact or laboratory samples, *after* the COVID-19 vaccine was available?	☐ Yes<br>☐ No
9. Did you attend any teaching session or meeting* that discussed COVID-19 Information and Research?<br>**includes any professional meetings organised or held outside NCID*	☐ Yes<br>☐ No
**Section B: Prior to COVID-19 Vaccine Availability**	
1. Were you afraid of getting COVID-19?	☐ 1: Not afraid at all<br>☐ 2: A bit afraid<br>☐ 3: Afraid<br>☐ 4: Very afraid
2. Did anyone you know (including family, friends, patients) have severe* COVID-19 disease?<br>[*Severe infection refers to needing to be admitted to ICU or on oxygen supplementation in hospital]	☐ Yes<br>☐ No

3. What was your reaction when COVID-19 vaccines were made available in Singapore?	☐ Overall Positive<br>☐ Overall Negative<br>☐ Comments (if any): _________
4. What was your reaction when you heard that frontline workers would have priority access to COVID-19 vaccines?	☐ Overall Positive<br>☐ Overall Negative<br>☐ Comments (if any): _________
5. Did you have any reservation about getting COVID-19 vaccinations?	☐ Yes (Proceed to Question B6)<br>☐ No (Proceed to Question B10)
6. What were your reservation(s) about the COVID-19 vaccination?	My reservations are:
7. You have some reservation about the COVID-19 vaccination. **Were you vaccinated eventually?**	☐ Yes (Proceed to Question B9)<br>☐ No (Proceed to Question B8)
8. Why did you choose not to be vaccinated? [Choose all that apply]<br><br>Skip Questions B9 to B12.<br>Proceed to Question B13.	☐ Afraid of injections<br>☐ I do not think the COVID-19 vaccine is effective<br>☐ I do not think the COVID-19 vaccine is safe<br>☐ COVID-19 is not a serious illness<br>☐ Concerned of medical complications (to self)<br>☐ Concerned of impact on fertility<br>☐ Concerned of impact on my child (breastfeeding, pregnant)<br>☐ Medically ineligible<br>☐ Others (please specify): ________
9. You have some reservation about the COVID-19 vaccination initially and proceeded to be vaccinated.<br><br>What made you change your mind? [Choose all that apply]	☐ Fear of illness / disease<br>☐ Confidence in drug approval process / safety profile<br>☐ Information: better understanding of vaccines<br>☐ Pressure from workplace<br>☐ Pressure from friends<br>☐ Pressure from family<br>☐ Vaccine differentiated measures (VDS)<br>☐ Others (please specify): ________
10. What motivated you to be vaccinated? [Choose all that apply]	☐ Fear of illness / disease<br>☐ Confidence in drug approval process / safety profile<br>☐ Information: better understanding of vaccines<br>☐ Pressure from workplace<br>☐ Pressure from friends<br>☐ Pressure from family<br>☐ Vaccine differentiated measures (VDS)<br>☐ Others (please specify): ________
11. When did you register yourself for your COVID-19 vaccination (first dose)?	☐ < 1 week from eligibility<br>☐ 1 week - 1 month from eligibility<br>☐ 1-3 months from eligibility

	☐ 3-6 months from eligibility<br>☐ >6 months from eligibility<br>☐ NA: I am unvaccinated / not planning on getting vaccinated / medically contraindicated
12. When did you register yourself for COVID-19 vaccination (booster dose)?	☐ < 1 week from eligibility<br>☐ 1 week - 1 month from eligibility<br>☐ 1-3 months from eligibility<br>☐ 3-6 months from eligibility<br>☐ >6 months from eligibility<br>☐ NA: I am unvaccinated / not eligible for a booster / not planning on getting boosted
13. Did NCID do anything to improve your understanding of the safety and efficacy of COVID-19 vaccinations prior to the vaccine roll out?	☐ Yes<br>☐ No<br>☐ Comments (if any): ________
14. Where did you get your information on COVID-19 vaccinations from? (select all that apply)	☐ Friends or family<br>☐ Local news sources (Straits Times, CNA, Mothership)<br>☐ Social Media (Facebook, TikTok, Instragram, Telegram group chats etc).<br>☐ Government or official sources of information<br>☐ Scientific Journals or other academic sources<br>☐ Hospital teaching and sharing<br>☐ Industry or professional sources (official communications, MOH Circulars)
15. Do you regularly assess sources of information for bias and accuracy?	☐ Yes<br>☐ No
16. Do you feel confident to assess sources of information for bias and accuracy?	☐ Yes<br>☐ No
17. Have you attended any talks or engagement sessions regarding COVID-19 vaccines?	☐ Yes, it was helpful<br>☐ Yes, but it was not helpful<br>☐ No
18. Would you accept receiving **yearly** COVID-19 booster vaccinations, if necessary? Why?	☐ Yes<br>☐ No<br>☐ Why: ________________
19. Would you accept receiving COVID-19 **variant specific booster vaccinations**, if necessary? Why?	☐ Yes<br>☐ No<br>☐ Why: ________________
End of questionnaire.<br><br>***Thank you for taking the time to participate in this survey.***	
